# Long-Term Nasolabial Appearance Post-Unilateral Cleft Lip Repair in a Single Center: A Descriptive Study

**DOI:** 10.7759/cureus.41683

**Published:** 2023-07-11

**Authors:** Muath Mamdouh Mahmod Al-Chalabi, Wan Azman Wan Sulaiman, Ahmad Sukari Halim

**Affiliations:** 1 Reconstructive Sciences Unit, Universiti Sains Malaysia (USM), Kota Bharu, MYS

**Keywords:** cleft lip and palate, unilateral cleft lip and palate, unilateral cleft lip, nasolabial appearance, cleft lip surgery

## Abstract

Introduction

Modern treatments still aim to keep the impact of surgical intervention low and the outcome of surgeries as good as a surgeon can. Assessing the long-term nasolabial appearance of patients who underwent cleft lip (CL) repair surgery is one of the methods of evaluating the outcomes of cleft surgery.

Methods

This is a retrospective cross-sectional descriptive study of data records of unilateral CL patients. The data records of all patients who underwent unilateral CL repair by the reconstructive science unit at Hospital Universiti Sains Malaysia (HUSM) within the first two years of their lives and whose current age is 14 years or above were accessed and analyzed.

Results

The data records of 50 patients were analyzed, including 13 (26%) males and 37 (74%) females. The surgeons opined that 28% of the patients had an acceptable nasolabial appearance, while there were 10 (20%) patients whose nasolabial appearance was considered unacceptable by the reviewing surgeons. Fifteen (30%) patients were described as having an acceptable lip appearance with secondary nasal deformity, and 11 (22%) patients had an acceptable nasal appearance with secondary lip deformity. There were no surgical modifications or postoperative complications among the patients. None of our variables reported a significant association with long-term nasolabial appearance.

Conclusion

The long-term evaluation of the nasolabial appearance in individuals with CL following surgical correction significantly improves the service and care provided to patients to achieve optimum results. Although our results showed no relationship between gender, age at operation, type or diagnosis of cleft, and family history and long-term nasolabial appearance, frequent assessments will enhance surgical results.

## Introduction

The cleft lip and palate (CLP) or cleft lip and alveolus (CLA) is a congenital facial deformity malformation that affects both function and aesthetics due to the absence of a union of the palatine processes throughout embryonic life. It is present in one in 700-1,100 births worldwide [1-3] and is considered one of the most common craniofacial anomalies. However, the prevalence varies according to race or ethnicity, sex, and type of cleft [4]. Clinically, the clefts can be found unilaterally or bilaterally, with unilateral clefts being the most frequent [3].

Usually, children born with cleft lips (CL) undergo a series of corrective surgeries [5,6] within the first 24 months of life [6], and this may be prolonged over many years [5]. Typically, recommendations for lip repair were made by the plastic surgeon, and consultations occurred with the orthodontist and oral surgeon concerning the desired aesthetic consequences of the surgery and the need for and timing of bone grafting procedures if needed [7]. The Millard rotation advancement repair has remained the most preferred procedure among cleft surgeons ever since it was first described. However, most plastic surgeons use modified techniques [8]. Cleft lip (CL) surgery is one of the most dramatic surgical procedures to make a disfigured face acceptable. There is evidence that the plastic surgeon's skill may be a more important influence on the outcome than the timing or technique used for CL repair [9].

The purpose of the surgical procedure is not limited to reconstructing the anatomy of the nasolabial region but extends to enhancing the patient's facial aesthetics and function [10]. Perfections in the appearance of the lip and nose are the most commonly desired aspects for further treatment by patients with clefts [11]. It needs to be known that nasolabial proportions and symmetry may still be subject to change during facial growth [12].

As modern treatments and surgical attempts still aim to keep the number and impact of scars associated with the surgical intervention low and the outcome of surgeries as good as the surgeon can, this study was conducted on the same track to achieve a good evaluation of long-term lip repair surgery outcomes, particularly nasolabial appearance. This study looks for long-term outcomes (particularly nasolabial appearance) in patients who underwent unilateral CL repair surgery by the reconstructive science unit in Hospital Universiti Sains Malaysia (HUSM) after almost completing their facial bone growth.

Puberty typically begins between 10 and 12 years in females and 12 and 14 years in males. This period is characterized by an enhanced growth rate that peaks approximately two years after puberty. Mid-facial projection also reaches maturity at 14 years in males and 13 years in females [13]. Generally, the face matures between 12 and 15 years old in males and two years earlier in females [14]. Therefore, we used the word "long-term" to emphasize that we are looking for the result after the age of 14 when the facial bone growth is completed, which gives almost the final appearance.

## Materials and methods

This is a retrospective cross-sectional study of data records of patients who had unilateral CL repair performed by the plastic and reconstructive science unit in HUSM to determine the long-term nasolabial outcome. Institutional ethical approval was obtained from our institutional review board. The study area is HUSM. The study population includes all non-syndromic patients who underwent unilateral CL repair by the plastic and reconstructive science unit in HUSM within the first two years of their lives and whose age is 14 years or above. Patients with incomplete data records or those who underwent lip repair in another center and subsequently continued follow-up with the plastic reconstructive science unit in HUSM are excluded from this study.

Throughout this study, the sampling method and subject recruitment included all patients who met the inclusion criteria. Their medical records were accessed and revised to fill out the proforma. Only case notes from the archive were revised, and proformas were filled in accordingly. The Statistical Package for the Social Sciences (SPSS) version 24 (IBM SPSS Statistics, Armonk, NY, USA) was used to analyze the data. Continuous data were summarized using descriptive and inferential statistics.

## Results

One hundred ninety patients were operated on between 1997 and 2008, but only 50 fulfilled the inclusion criteria when reviewing the data records. Regarding demographical details, the total number of patients was 50, representing 37 female and 13 male patients. Table 1 shows the demographic details.

**Table 1:** Demographic details of patients. n = number of patients, CLP = cleft lip and palate, CLA = cleft lip and alveolus, CL = cleft lip

Variable	n	%
Gender		
Male	13	26
Female	37	74
Age at operation (months)		
3	24	48
4	14	28
5	7	14
6	2	4
7	1	2
13	1	2
15	1	2
Cleft diagnosis		
Left incomplete CLP	5	10
Left incomplete CLA	1	2
Left incomplete CL	2	4
Left complete CLP	27	54
Right incomplete CLP	0	0
Right incomplete CLA	1	2
Right incomplete CL	4	8
Right complete CLP	10	20
Family history of cleft		
No	35	70
Yes	15	30
Presence of secondary deformity		
None	13	26
Lip	9	18
Nose	13	26
Both	15	30
Revision surgery		
No	13	26
Yes	12	24
Twice	3	6
Pending	22	44
Surgeon's comment on nasolabial appearance		
Acceptable nasolabial appearance	14	28
Acceptable nasal appearance with secondary lip deformity	11	22
Acceptable lip appearance with secondary nasal deformity	15	30
Unacceptable nasolabial appearance	10	20

Most patients are female (74%) and operated on before the age of six months (90%). Clefts occur commonly on the left side (70%) and are the complete type (74%). The left complete cleft was the most common among all possible types of cleft (54%). Most patients have no family history of cleft lip (70%). The majority have secondary deformities (74%) on either the lip, nose, or both, with only 15 (30%) patients having revision surgery. The surgeons opined that 28% of the patients had an acceptable nasolabial appearance, while there were 10 (20%) patients whose nasolabial appearance was considered unacceptable by the reviewing surgeons. No surgical modifications or postoperative complications among patients affected the nasolabial outcome of unilateral cleft lip repair surgery.

Table 2 shows a surgeon's comments on the presence of the fine scar (which generally reflects a good outcome) or any secondary deformities with different variables.

**Table 2:** Cross-tabulation of surgeon's comment on lip scar appearance according to variables. CLP = cleft lip and palate, CLA = cleft lip and alveolus, CL = cleft lip

Variable	Surgeon's comment	
	Fine scar	Secondary deformity
Gender		
Male	10	3
Female	16	21
Age at operation (months)		
3	15	9
4	7	7
5	2	5
6	1	1
7	1	0
13	0	1
15	0	1
Cleft diagnosis		
Left incomplete CLP	3	2
Left incomplete CLA	1	0
Left incomplete CL	1	1
Left complete CLP	12	15
Right incomplete CLP	2	2
Right incomplete CLA	0	1
Right incomplete CL	0	0
Right complete CLP	7	3
Family history of cleft		
No	19	16
Yes	7	8

The surgeon commented that a more significant proportion of females (56.8%) had secondary lip deformity compared to male (23.1%) patients. In addition, patients who were operated on before seven months of age have either a fine lip scar or a secondary lip deformity. In contrast, those operated on at 13 and 15 months only have a secondary lip deformity. Regarding the cleft type or diagnosis, all patients of different types have fine scars or secondary lip deformities except for left and right incomplete CLA. The proportion of those with fine lip scars and secondary lip deformities seems equally distributed among those with or without a family history of the cleft.

## Discussion

Our demographic data showed female predominance in patients with a unilateral cleft, which is compatible with what Shah et al. [15] reported in their study as they assessed demographic data on the characterization of oral clefts in Malaysia. They reported that females were more involved in oral cleft conditions than males (56.7% and 43.3%, respectively). We also reported more female involvement than males (74% and 26%, respectively), emphasizing female predominance in Malaysia, possibly due to females being more concerned about their follow-up. In contrast, other European studies reported male predominance, as Vlastos et al. [16] mentioned in a retrospective study of the records of all children with cleft lip or palate treated within the Centre of Craniofacial Anomalies of Aghia Sophia Children Hospital of Athens from 1995 to 2007. In addition, Mani et al. [17] also reported male predominance in Sweden. On the other hand, Sharif et al. [18] in Pakistan, Al Omari et al. [4] in Jordan, Jamilian et al. [19] in Iran, and Aljohar et al. [20] in Saudi Arabia reported male predominance. Sinko et al. [2] and Mani et al. [21] reported that female faces tend to get better nasolabial scores in the long term than male faces, while Thompson et al. [22] reported no difference in nasolabial outcomes based on sex, which is compatible with our results.

The timing of CL repairs remains an area of controversy. Campbell et al. [23] mentioned that each cleft team advocates a somewhat different timing for lip repair, with the actual correction being performed throughout the period from neonate to six months or later. Accordingly, most lip repair operations in our study group were performed before six (90%). According to the results, Ziak et al. [24] concluded that lip closure in 3-6 months, including nasolabial appearance, is considered adequate. However, our results showed no significant association between the child's age at the operation and long-term nasolabial appearance or outcome.

CL is an entity with different variations. Our result showed that clefts occur commonly on the left side (70%), and the left complete cleft was the most common among all possible types of clefts (54%). This result is quite similar to the results reported by Shah et al. [15] in Malaysia, Al Omari et al. [4] in Jordan, and Abdurrazaq et al. [25] in Nigeria. Another study conducted in Pakistan by Elahi et al. [26] reported that the left side was more commonly involved in cleft lip-related anomalies, and cleft lip alone was observed more frequently than combined CLP or isolated cleft palate. Thompson et al. [22] found notable differences in nasolabial appearance among those with CL, while those with CLP had the worst outcomes. On the other hand, they reported no difference based on the sides (right or left) in the unilateral cleft. In this study, although most cleft types were left-sided with complete CL, the type does not appear related to long-term nasolabial appearances.

Recently, Bartzela et al. [27] published a study of the clinical characteristics of 266 patients and family members with CL and/or palate with associated malformations and syndromes in which they found that patients with CLA have a 26% positive family history in comparison to patients with CLP who have a 29% positive family history. Our result is very close to these percentages, as we reported that 30% of our patients have a positive family history. In the previous study, Aljohar et al. [20] reported that 31.9% of cleft patients have a positive family history. Two decades earlier, Elahi et al. [26] reported that only 17% of the patients had a positive family history of cleft anomalies. In the literature, there is no evidence of a relationship between family history and long-term nasolabial outcomes, and our results also did not show any significant association.

Regarding long-term nasolabial appearance, the results varied from an acceptable nasolabial appearance in 14 (28%) patients, while there are still 10 (20%) patients whose nasolabial appearance is considered unacceptable by the reviewing surgeons. Fifteen (30%) patients were described as having an acceptable lip appearance with secondary nasal deformity, while 11 (22%) patients had an acceptable nasal appearance with secondary lip deformity. This different percentage of long-term nasolabial appearance reflects the importance of frequent evaluation of surgical techniques and outcomes to improve the quality of service and care provided to the patients. Variation also confirms that the most challenging aspect of CL repair is the postoperative results or outcomes, especially long-term outcomes when facial bone growth is complete, which determines the ultimate facial appearance. Consequently, the treatment of clefts should involve surgical closure of the cleft and an aesthetically and functionally perfect result in adulthood [28].

In this study, we assessed long-term scar appearance, which got a comment from different plastic surgeons who usually attend the plastic and reconstructive outpatient clinic and follow-up on CL patients. Based on gender differences, we found a more significant proportion of females (56.8%) commented on by the surgeon as having a secondary lip deformity compared to males (23.1%). This may raise the question of whether the surgeons are more rigorous in accepting the scar appearance in females than in males or not. In contrast to our findings, Zhang et al. [29] reported that 10.6% of male patients had secondary lip deformities compared to 8.4% of female patients. Interestingly, our data showed that patients who were operated on before seven months old had either a fine lip scar or a secondary lip deformity, while those operated on at 13 and 15 months only had secondary lip deformities, suggesting that early operation reduces the chance of secondary deformities. Hammoudeh et al. [30] generally commented in their study that the earlier CL repair could be done safely and effectively to improve postoperative nasal symmetry. Regarding the side of the cleft associated with more secondary deformities, our results corresponded with Zhang et al. [29], who reported more association with left-sided clefts with a predominance of left complete CLP. The results also showed an equal chance of developing secondary deformity or not in the presence of family history.

However, the limited sample size of this study (due to a high exclusion rate) is one of its weaknesses. Additionally, many variables that need to be evaluated concerning long-term nasolabial appearance can be postulated. Last but not least, this study was done during the COVID-19 outbreak, which caused many difficulties.

## Conclusions

Orofacial clefts remain essential in most plastic and reconstructive surgical repairs worldwide. An effective evaluation of the outcomes, particularly the long-term appearance of the nasolabial complex, plays a significant role in improving the service and care provided to patients to achieve optimum results. Our results showed no relationship between gender, age at operation, type or diagnosis of the cleft, family history, and long-term nasolabial appearance. Still, such a follow-up may determine any complications or factors affecting long-term outcomes.
